# MRI-guided in-bore biopsy of the prostate – defining the optimal number of cores needed

**DOI:** 10.1186/s40644-024-00734-3

**Published:** 2024-07-01

**Authors:** Moritz Gross, Edith Eisenhuber, Petra Assinger, Raphael Schima, Martin Susani, Stefan Doblhammer, Wolfgang Schima

**Affiliations:** 1 Department of Diagnostic and Interventional Radiology, Goettlicher Heiland Krankenhaus, Barmherzige Schwestern Krankenhaus, and Sankt Josef Krankenhaus, Dornbacher Strasse 20-30, Vienna, 1170 Austria; 2 Medical University of Vienna, Wien, Austria; 3 Varga, Braun, Pathology Laboratory, Vienna, 1210 Austria; 4 Department of Urology, Krankenhaus Korneuburg, Korneuburg, Austria

**Keywords:** Prostate biopsy, MRI-targeted, In-bore, In-gantry, Number of cores

## Abstract

**Background:**

Numerous studies have shown that magnetic resonance imaging (MRI)-targeted biopsy approaches are superior to traditional systematic transrectal ultrasound guided biopsy (TRUS-Bx). The optimal number of biopsy cores to be obtained per lesion identified on multiparametric MRI (mpMRI) images, however, remains a matter of debate. The aim of this study was to evaluate the incremental value of additional biopsy cores in an MRI-targeted “in-bore”-biopsy (MRI-Bx) setting.

**Patients and methods:**

Two hundred and forty-five patients, who underwent MRI-Bx between June 2014 and September 2021, were included in this retrospective single-center analysis. All lesions were biopsied with at least five biopsy cores and cumulative detection rates for any cancer (PCa) as well as detection rates of clinically significant cancers (csPCa) were calculated for each sequentially labeled biopsy core. The cumulative per-core detection rates are presented as whole numbers and as proportion of the maximum detection rate reached, when all biopsy cores were considered. CsPCa was defined as Gleason Score (GS) ≥ 7 (3 + 4).

**Results:**

One hundred and thirty-two of 245 Patients (53.9%) were diagnosed with prostate cancer and csPCa was found in 64 (26.1%) patients. The first biopsy core revealed csPCa/ PCa in 76.6% (49/64)/ 81.8% (108/132) of cases. The second, third and fourth core found csPCa/ PCa not detected by previous cores in 10.9% (7/64)/ 8.3% (11/132), 7.8% (5/64)/ 5.3% (7/132) and 3.1% (2/64)/ 3% (4/132) of cases, respectively. Obtaining one or more cores beyond the fourth biopsy core resulted in an increase in detection rate of 1.6% (1/64)/ 1.5% (2/132).

**Conclusion:**

We found that obtaining five cores per lesion maximized detection rates. If, however, future research should establish a clear link between the incidence of serious complications and the number of biopsy cores obtained, a three-core biopsy might suffice as our results suggest that about 95% of all csPCa are detected by the first three cores.

**Supplementary Information:**

The online version contains supplementary material available at 10.1186/s40644-024-00734-3.

## Introduction

For decades, the detection of PCa solely relied on systematic transrectal ultrasound guided biopsy. This approach utilizes transrectal ultrasound (TRUS) to guide the biopsy needle to 10–12 predetermined regions of the prostate, from where tissue samples are then taken. However, since TRUS is unable to reliably detect PCa [1], TRUS-Bx remains a random sampling technique. This results in three main problems. Firstly, and not surprisingly, TRUS-Bx misses a considerable number of clinically significant cancers [2, 3]. Secondly, random sampling reveals clinically insignificant cancers (ciPCa), most frequently defined by the ‘Epstein’ criteria (Gleason Score 6 [3 + 3], less than 3 cores with cancer involvement, no core with cancer involvement ≥ 50% and a prostate-specific-antigen-density of < 0.15 ng/ml^2^), which are unlikely to cause any symptoms in the patient’s lifetime, but might contribute to subsequent overtreatment once being diagnosed [4]. Lastly, TRUS-Bx tends to misclassify tumors with a substantial number of tumors being upgraded on final pathology [5]. This, in turn, can lead to incorrect treatment decisions being made and might have serious consequences, especially as new therapeutical options are getting more target-specific and some treatments are only available to certain risk groups [6].

Due to the clinical implementation of multiparametric magnetic resonance imaging, detection of csPCa is now possible with high sensitivity [7]. Multiparametric MRI combines anatomical MRI sequences with functional sequences to find lesions within the prostate that are suspicious of cancer. These lesions or regions of interest (ROIs) can then be biopsied in an MRI-targeted biopsy approach. Compared to TRUS-guided biopsy, this strategy demonstrates improved detection rates (DR) of csPCa, as well as a reduction in the DR of ciPCa and a reduction of up- or down-gradings at final pathology [8, 9]. In addition, MRI-targeted biopsy procedures can establish a diagnosis using only a fraction of the number of cores needed in TRUS-guided biopsy.

With cognitive fusion biopsy (COG-Bx), MRI-TRUS fusion biopsy (FUS-Bx) and MRI-Bx, three techniques to perform MRI-targeted biopsies have evolved in recent decades. COG-Bx and FUS-Bx also rely on TRUS to direct the biopsy needle to the target’s location, whereas MRI-Bx uses near real-time mpMRI images for targeting. Although each technique has their advantages and disadvantages, the question, whether there is an optimal technique, remains a controversial subject in different studies comparing these techniques [10, 11]. Intuitively, MRI-Bx would be the most accurate technique since it is the only technique that offers direct visualization of the lesion during the biopsy procedure. Evidence supporting this theory comes from recent retrospective studies by Costa et al. and Prince et al. [12, 13]. Costa et al. compared the results of 300 men that underwent FUS-Bx with the results of 103 men in the MRI-Bx-arm of the study and found that MRI-Bx detected more csPCa and fewer ciPCa than FUS-Bx. Both results were statistically significant [12]. Prince et al. compared the target-specific cancer detection of FUS-Bx and MRI-Bx and found a significantly higher likelihood of detecting any PCa with MRI-Bx compared to FUS-Bx. The likelihood of detecting csPCa was also in favor of MRI-Bx, although this result was not statistically significant [13]. On the other hand, FUS-Bx can be combined with random TRUS-Bx in one session, which will increase the overall number of cancers detected [14]. To answer the question for the best method of guided biopsy, more research is needed.

While the benefits the MRI-targeted approach offers are well studied, the optimal number of biopsy cores obtained per lesion remains elusive. Factors impacting this number are the detection rates of PCa and csPCa in particular, as well as post-procedural complication rates, which seem to correlate with the number of cores taken [15]. Studies addressing this issue are scarce and most are conducted in a FUS-Bx setting. To the best of our knowledge, there are only three studies that analyzed the optimal number of cores in the context of MRI-Bx [16–18]. In addition, the biopsy protocols in these studies consisted of 2–3 cores (as a default, in some cases more) per ROI only, which means that there are almost no data on potential benefits of routinely obtaining additional cores beyond the third biopsy core in MRI-Bx.

For these reasons, we believe that more research is needed to define the optimal number of cores to be obtained per ROI in an MRI-Bx setting. The MRI-Bx protocol at our institution consists of five sequential cores per lesion that are obtained in a systematic manner. The aim of this retrospective single center study was to analyze the effect each of the cores obtained had on csPCa and PCa detection and to answer the question if patients would benefit from increasing the number of biopsy cores in MRI-Bx to five cores per ROI.

## Patients and methods

### Patients

**Fig. 1:**
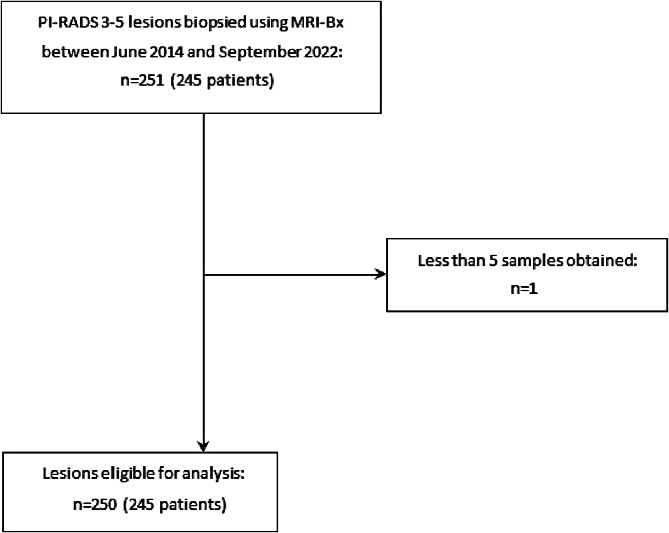
Flowchart for patient acquisition

This retrospective single-center study was approved by our institutional review board and informed consent was waived. All eligible patients who underwent MRI-Bx upon suspicion of prostate cancer at our institution between June 2014 and September 2021 were included. A lesion was deemed suspicious if it received a score of Prostate Imaging – Reporting and Data System (PI-RADS) ≥ 3. Since PI-RADS guidelines were updated two times between 2014 and 2021, patients who underwent mpMRI in the years 2014–2016 were graded using PI-RADS v.1 [19], patients who underwent mpMRI in the years 2016–2019 were graded using PI-RADS v.2 [20] and all patients after 2019 were graded using PI-RADS v.2.1 [21]. Lesions, which had been biopsied with less than 5 cores, were excluded from the analysis. In total 245 patients with 250 lesions biopsied met eligibility criteria (Fig. 1). One hundred forty-two patients were included in a prior study that evaluated the diagnostic performance, complication rates and the learning curve of MRI-Bx [22].

### Diagnostic mpMRI

Diagnostic mpMRI was performed either at our institution or mpMRI studies obtained elsewhere were re-analyzed. In case mpMRI images were not obtained at our institution the images were carefully examined by a radiologist at our institution with at least 20 years of body MRI experience to ensure high quality of the images and confirm indication for the procedure. MpMRI at our institution was conducted in a 3.0T MRI scanner (Magnetom Skyra 3T, Siemens, Erlangen, Germany) using an 18-channel body array coil (Body 18, Siemens). The MRI-protocol consisted of T1- and T2-weighted (T1w and T2w) images in combination with diffusion-weighted images (DWI) with apparent diffusion coefficient (ADC) mapping and dynamic contrast-enhanced images (DCE). The detailed protocol is provided in Table 1. Image analysis was performed by one of three radiologists (5–20 years of body MRI experience) at our institution according to the latest PI-RADS guidelines.

**Table 1:** Pulse sequence parameters of mpMRI

	ax. T1w VIBE DIXON	ax. T2w TSE	cor. T2w TSE	sag. T2w TSE	ax. DWI b0, 1500	ax. gado- T1w VIBE dyn.	ax. gado- T1w VIBE post
TR (ms)	5.7	7500	7500	7870	4940	4	4
TE (ms)	2.5	99	113	94	59	1.3	1.3
Flip angle (degrees)	9	160	160	160	180	9	9
Fat saturation	Yes + no	No	No	No	No	Yes	Yes
Slice thickness (mm)	3	3.5	3.5	3.5	3.5	3	3
Gap (mm)	0	0	0	0	0	0	0
Matrix	320 × 208	384 × 384	320 × 320	320 × 310	106 × 106	320 × 195	320 × 208
FOV (mm)	350 × 350	199 × 199	220 × 220	240 × 240	300 × 300	308 × 280	284 × 350
Acquisition time (min: sec)	0:45	4:00	2:52	3:24	8:55	5:26	0:46

Ax. = axial, VIBE = Volumetric interpolated breath-hold examination, TSE = Turbo spin echo, cor. = coronal, sag. = sagittal, DWI = Diffusion weighted imaging, TR = Repetition Time, TE = Time to Echo, FOV = Field of View

### mpMRI and biopsy procedure

**Fig. 2:**
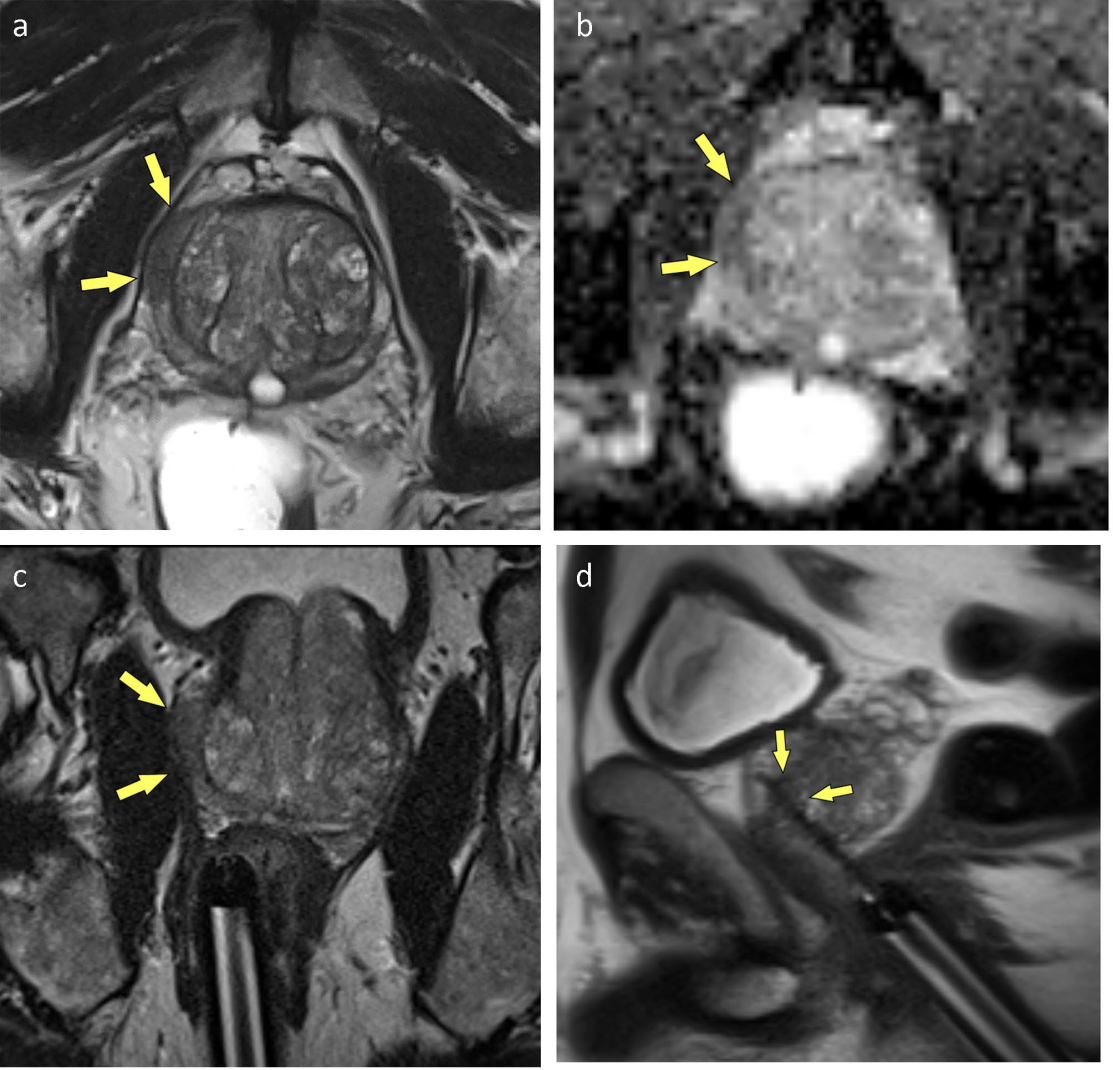
(**a-d**) PI-RADS 5 lesion in the PZ (arrows). The tumor was initially classified as ISUP grade group 1 but was eventually upgraded core by core, resulting in its classification as an ISUP grade group 5 tumor. (**a**) T2w, (**b**) ADC, (**c**) needle-guide aimed at lesion, (**d**) confirmatory scan with needle in situ

**Fig. 3:**
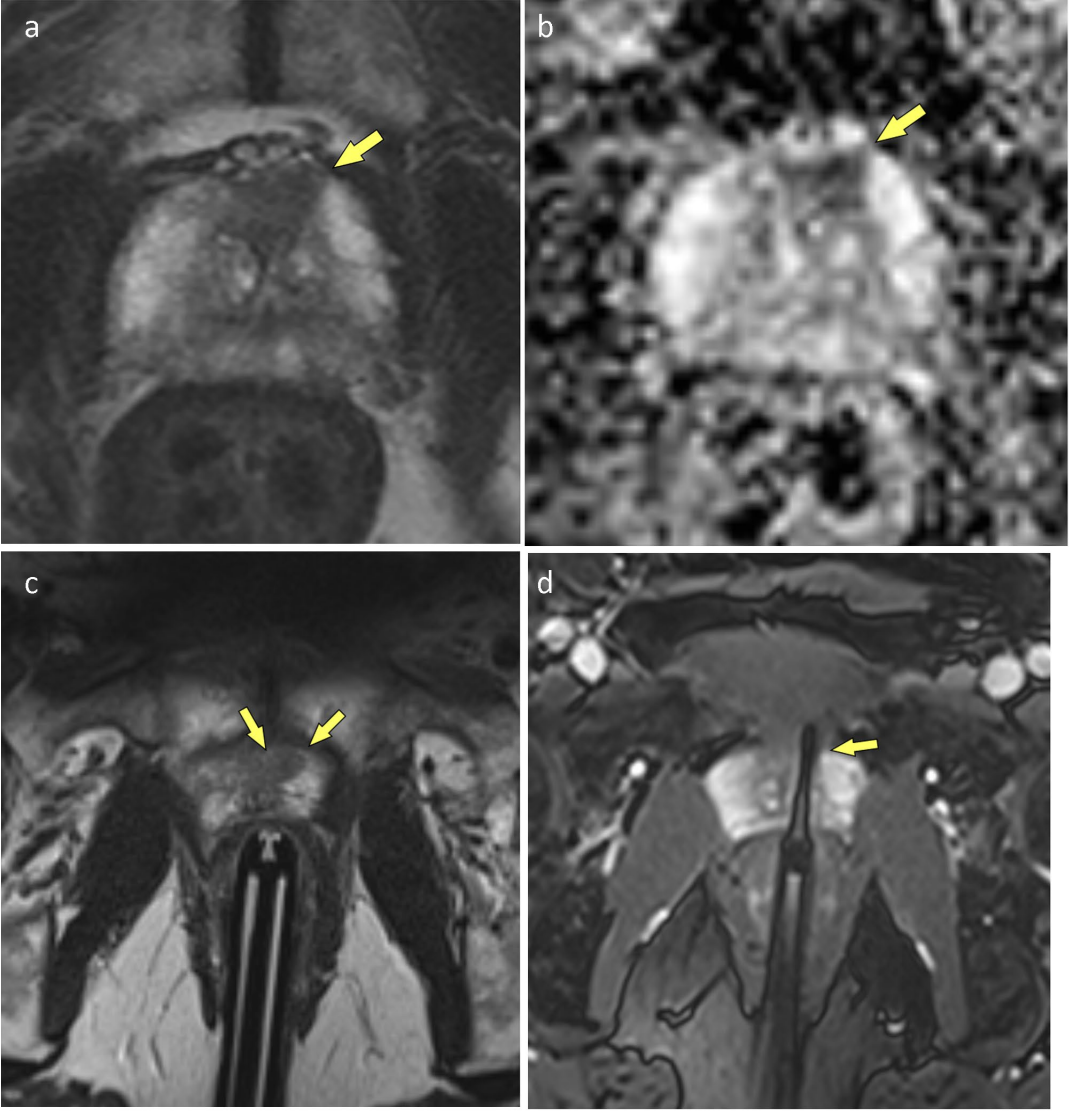
(**a-d**) PI-RADS 4 lesion in the TZ/AS (arrows). This tumor was initially classified as ISUP grade group 3 by the first biopsy core and was eventually upgraded to an ISUP grade group 5 tumor. (**a**) T2w, (**b**) ADC, (**c**) needle-guide aimed at lesion, (**d**) confirmatory scan with needle in situ

All biopsies were performed in a 3-Tesla MRI-Scanner (Magnetom Skyra 3T, Siemens, ) using an 18-channel array body coil (Body 18, Siemens). MR protocol comprised axial T2w TSE and DWI sequences for lesion location and axial and sagittal obliques T2w TSE for needle guide alignment (Table 2). Antibiotic prophylaxis was conducted with fluroquinolones (ciprofloxacine 500 mg peroral, Ciproxin^®^, Bayer AG, Leverkusen, Germany) and started the evening before the biopsy to be continued for another 4 days. In case of fluoroquinolone allergy or resistance amoxicillin/clavulanic acid (Augmentin^®^, GlaxoSmithKline, London-Brentford, UK) was used instead. Patients were administered 10 mg of nalbuphine IV (Nalbuphin Amomed, Amomed Pharma GmbH, Vienna, Austria) right before the procedure and 2% lidocaine gel was applied to the needle-guide (Invivo, Gainesville, FL, USA [now Philips, Amsterdam, Netherlands]) for analgesia. The patient was placed in prone position with the hollow needle-guide placed in the patient’s rectum. The needle-guide was than attached to the DynaTrim clamp stand (Invivo) and mpMRI images (axial T2w TSE, DWI) to locate the lesion again were obtained. Correct placement of the needle-guide was achieved by applying adjustment parameters provided by the DynaCAD Software (Invivo) to the DynaTrim device. After ensuring the needle guide was properly aligned with the ROI the first sample was taken (Fig. 2c/Fig. 3c).

**Table 2:** Pulse sequence parameters of MR-guided in-bore biopsy

	sag. T2w HASTE	ax. T2w TSE	sag. T2w TSE	ax. DWI b0, 1500	paraax./ parasag. T2w TSE	paraax. TruFISP
TR (ms)	1400	7500	7500	4640	6500	690
TE (ms)	101	101	101	78	96	1.8
Flip angle (degrees)	126	160	160	180	160	50
Fat saturation	No	No	No	No	No	Yes
Slice thickness (mm)	3	3	3	3	3	3
Gap (mm)	0	0	0	0	0	0
Matrix	320 × 256	320 × 288	320 × 288	112 × 112	320 × 310	256 × 175
FOV (mm)	300 × 300	220 × 220	240 × 240	220 × 220	300 × 300	300 × 300
Acquisition time (min: sec)	0:22	2:52	2:52	3:58	0:59	0:22

Sag. = sagittal, HASTE = Half-Fourier Acquisition Single-shot Turbo spin Echo, ax. = axial, TSE = Turbo spin echo, DWI = Diffusion weighted imaging, paraax. = paraxial, parasag. = parasagittal, TruFISP = True fast imaging with steady-state free precession, TR = Repetition Time, TE = Time to echo, FOV = Field of view

**Fig. 4:**
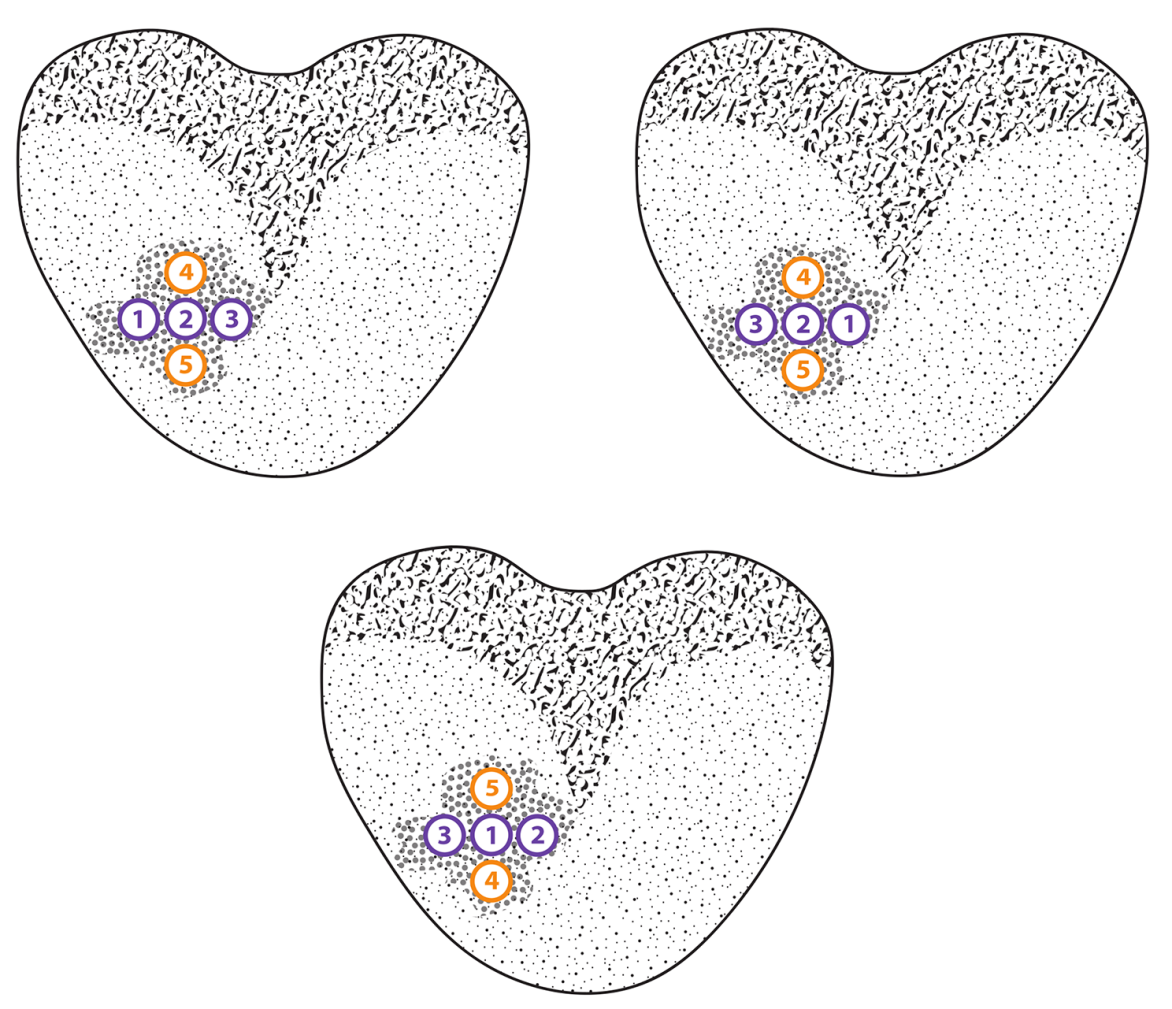
Sequential order and distribution of biopsy cores obtained

In accordance with our institutional protocol, each ROI was biopsied with at least five sequential biopsy cores with the initial core aimed at the geometrical center of the lesion. The second and third core were taken one angular degree to the left and one angular degree the right of the first core in coronal plane. The fourth and fifth core were then taken one angular degree above and below the position of the first biopsy core. More samples could have been obtained as to the biopsy operator’s discretion. All samples were kept in separate containers and labelled sequentially. There was no directive regarding the exact order of the second and third or fourth and fifth core, respectively, but the second and third core had to be obtained in the same axial plane as the first core but lateral to it. The fourth and fifth core had to be obtained inferiorly and superiorly (or vice versa) to the first core (Fig. 4).

### Histopathological analysis

Each biopsy core was submitted in a separate jar that was exactly labelled according to the region of interest and the biopsy protocol. All biopsy specimens were graded in accordance with the International Society of Urological Pathology’s (ISUP) recommendations for Gleason Grading [23, 24]. Each biopsy core was reported on individually. The histopathological report comprised the number of biopsy cores taken, the total length of each core, as well as the amount of any Gleason pattern present in the core. As recommendations on reporting findings from mpMRI-guided biopsies changed after the ISUP consensus conference in 2019 and the publication of new recommendations by the Genitourinary Pathology Society (GUPS), every histopathological report produced thereafter also contained a global Gleason score taking the findings of all biopsy cores into account [24, 25]. For every biopsy procedure that took place before these recommendations could be implemented at our institution, a global Gleason score was calculated retrospectively.

### Outcome parameters

Primary outcome parameter was the cumulative effect of each additional biopsy core on the detection rate of csPCa. Secondarily, the cumulative effect of each additional core on overall PCa detection was analyzed.

### Statistical analysis

Statistical analysis was performed using the Python programming language (Python Software Foundation, Beaverton, USA). To determine the cumulative effect of each core, a global Gleason Score that takes all prior biopsy samples into account was calculated after the second, third and fourth core. Detection rates for csPCa and overall detection rate of PCa are given as frequencies and as percentage of the maximum detection rate (all biopsy cores considered). In addition, Wilson Score Intervals were calculated for the cumulative detection rates of each core.

All other descriptive statistics are given as mean and standard deviation or median and interquartile range (IQR) for continuous variables and frequencies and percentages for categorial variables.

## Results

Of 245 Patients with 251 lesions that were biopsied at our institution between June 2014 and September 2021, 245 Patients and 250 lesions met eligibility criteria. Five patients had a second biopsy of a newly found lesion sometime after their first biopsy procedure. Since parameters like PSA-value, age, PI-RADS score and lesion location differed between the first and second biopsy, the values at the time of the second biopsy were also considered when calculating the mean, standard deviation, median, interquartile range, or frequency of the aforementioned parameters. Detailed information on patient and lesion characteristics are provided in Table 3.

**Table 3:** Patient demographics and lesion characteristics

	Negative (%)	GG1 (%)	≥GG2 (%)	All (%)
N (lesions) N (patients)	118 (47.2%) (113 pat. [46.1%])	68 (27.2%) (68 pat. [27.8%])	64 (25.6%) (64 pat. [26.1%])	250 (245 pat.)
Age (mean ± SD, y)	64.8 ± 8.4	67.0 ± 7.7	70.0 ± 7.0	66.8 ± 8.1
PSA (ng/ml)	10.0 ± 9.5	9.1 ± 5.7	12.0 ± 13.6	10.3 ± 9.9
mpMRI PI-RADS:				
PI-RADS Score 3	10.2% (12/118)	4.4% (3/68)	1.6% (1/64)	6.4% (16/250)
PI-RADS Score 4	71.2% (84/118)	76.5% (52/68)	65.6% (42/64)	71.2% (178/250)
PI-RADS Score 5	18.6% (22/118)	19.1% (13/68)	32.8% (21/64)	22.4% (56/250)
Location:				
PZ	45.3% (58/128)	57.9% (44/76)	48.7% (38/78)	56.0% (140/250)
TZ	49.2% (63/128)	28.9% (22/76)	34.6% (27/78)	44.8% (112/250)
CZ	0.8% (1/128)	2.6% (2/76)	0% (0/78)	1.2% (3/250)
AS	4.7% (6/128)	10.5% (8/76)	16.7% (13/78)	10.8% (27/250)

PZ = peripheral zone, TZ = transition zone, CZ = central zone, AS = anterior fibromuscular stroma

Note: lesions may affect more than one zone

Most of the lesions were biopsied with 5 cores according to our institution’s protocol (86% [215/250]). Six, 7, 8, 9 and 10 cores were obtained in 11% (28/250), 0.8% (2/250), 0.8% (2/250), 0.8% (2/250) and 0.4% (1/250) of cases, respectively. In total 1301 biopsy cores were obtained. The median number of biopsy cores obtained per patient was 5, with an IQR of 0.

**Fig. 5:**
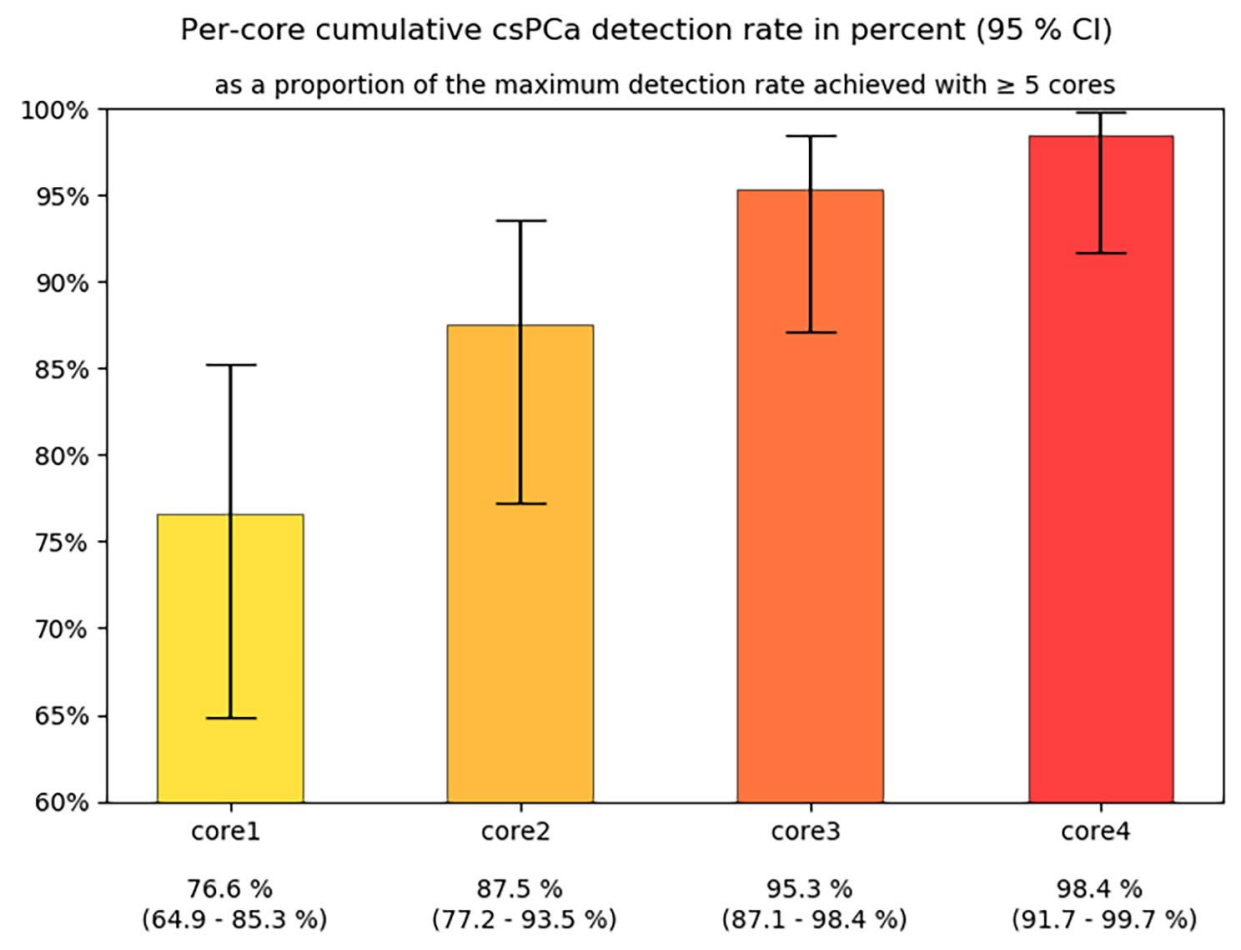
Per-core cumulative csPCa detection rate (%; 95% CI)

**Fig. 6:**
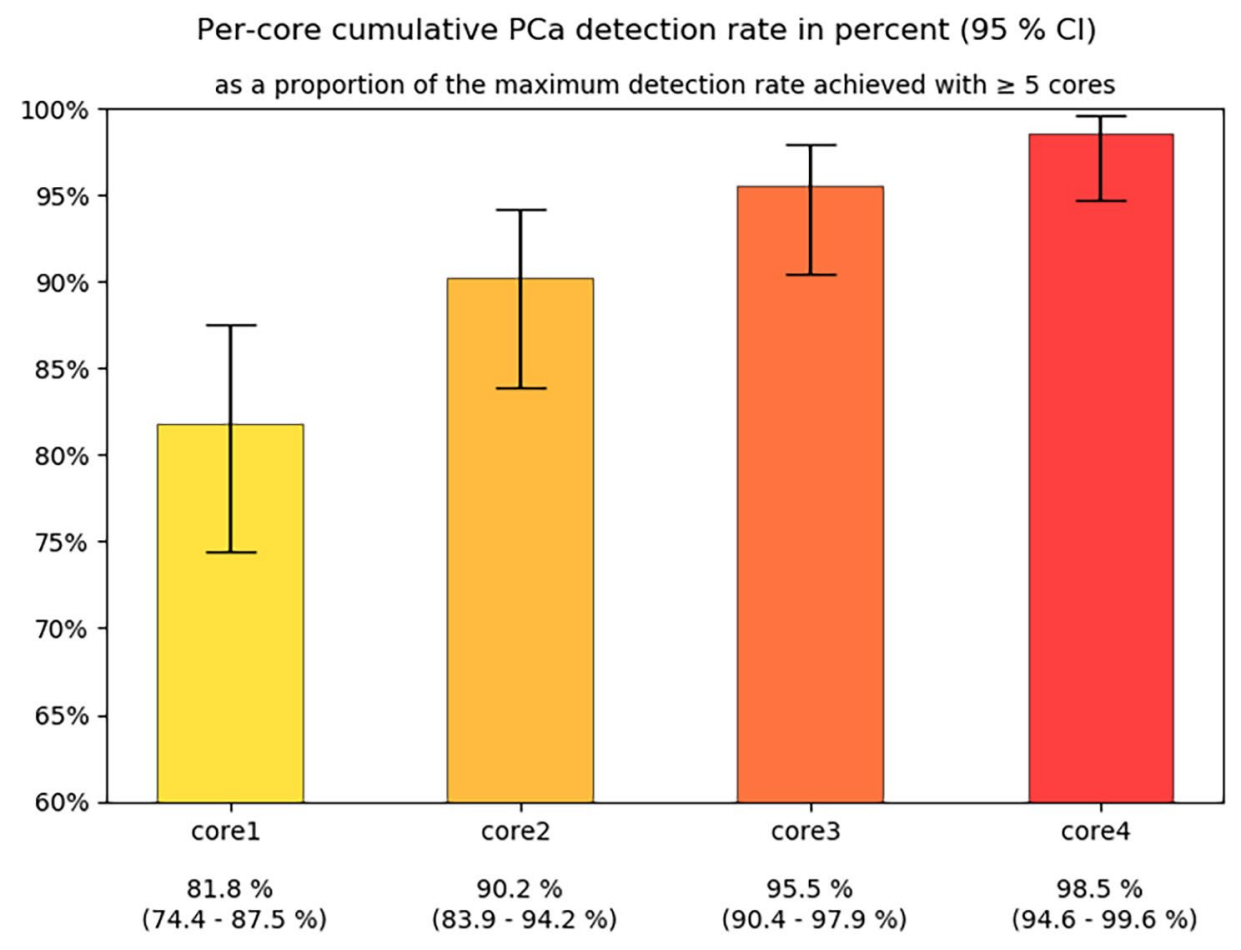
Per-core cumulative PCa detection rate (%; 95% CI)

Of 245 patients 132 (54%) were found to have PCa and 64 (26%) harbored csPCa. Detailed biopsy results are provided in Table 4. Of all csPCas diagnosed, 76.6% (49/64) were identified by the first biopsy core. The second, third and fourth core found csPCa not detected by previous cores in 10.9% (7/64), 7.8% (5/64) and 3.1% (2/64) of cases, respectively. Obtaining one or more cores beyond the fourth biopsy core resulted in an increase in detection rate of 1.6% (1/64) (Fig. 5). Results were similar, when clinically insignificant cancers were also considered. Of all cancers detected, the first biopsy core identified 81.8% (108/132). Adding the second, third and fourth core increased the detection rate by 8.3% (11/132), 5.3% (7/132) and 3% (4/132), respectively. Adding cores beyond the fourth increased the detection rate by 1.5% (2/132) (Fig. 6).

**Table 4:** Biopsy results on a lesion level

PCa, Gleason Score	ISUP Grade Group	*N* (%)
No PCa		118 (47.2%)
6 (3 + 3)	1	68 (27.2%)
7 (3 + 4)	2	31 (12.4%)
7 (4 + 3)	3	19 (7.6%)
8 (3 + 5)	4	1 (0.4%)
8 (4 + 4)		4 (1.6%)
9 (4 + 5)	5	5 (2.0%)
9 (5 + 4)		2 (0.8%)
10 (5 + 5)		2 (0.8%)

Of the 132 PCas detected 36.4% (48/132) would have been missed or misclassified by the first biopsy core (40 missed or upgraded, 8 downgraded). Upgrades as well as downgrades were limited to the next higher or lower grade group in most cases, although two tumors were upgraded from an ISUP grade group 3 to a grade group 5 and one ISUP grade group 1 tumor was gradually upgraded to a grade group 5 tumor core by core. In case of down-gradings, two tumors were downgraded to a grade group 2 from initially being classified as ISUP grade group 4.

Of all cancers detected by the first biopsy core, the addition of a second core resulted in an ISUP grade group upgrade/ downgrade in 9.3% (10/108)/ 4.6% (5/108), including one upgrade from grade group 3 to grade group 5 and one downgrade from grade group 4 to grade group 2. Adding a third core led to an upgrade/ downgrade in 4.2% (5/119)/ 3.4% (4/119). Adding a fourth or fifth (or n-th) core resulted in an upgrade/ downgrade in 3.2% (4/126)/ < 1% (1/126) and 2.3% (3/130)/ 1.5% (2/130), respectively. In one instance the addition of a fifth core led to an upgrade from ISUP grade group 3 to grade group 5.

## Discussion

Our data demonstrates that every additional biopsy core up to 5 cores (or more in rare cases) results in an increase in detection rate. This was true for overall PCa detection as well as the detection of csPCa. In both cases, this effect was more pronounced when the number of cores increased from one to three than from three to five. Increasing the number of cores from one to three resulted in an increase in detection rate of 13.6% (18/132) for any PCa and 18.8% (12/64) for csPCa, whereas increasing the number of cores from three to five (or more) resulted in an increase of only 4.5% (6/132) for any PCa and 4.7% (3/64) for csPCa.

This is not surprising considering that although mpMRI is highly sensitive for the detection of prostate cancer targeting inaccuracies might still occur due to needle deflection for example. Moreover, studies could show that the actual tumor size is often underestimated on mpMRI images [26] and most prostate cancers are histologically heterogeneous [27]. All these problems can only be met by increasing the number of cores and sampling an area larger than indicated by mpMRI findings.

Studies conducted in a COG-Bx or FUS-Bx setting showed results similar to ours. Zhang et al. retrospectively analyzed, how the number of biopsy cores affected the detection rates of PCa and csPCa in 330 patients that underwent COG-Bx as per their institution’s active surveillance protocol or for elevated PSA after negative initial biopsy. Zhang et al. found that by increasing the number of biopsy cores from one to three they detected 8.2% (27/330) and 6.4% (21/330) more PCa and csPCa, respectively. By increasing the number of cores from three to five detection rates increased by 3.3% (11/330) for PCa and 2.4% (8/330) for csPCa [28]. Tracy et al. analyzed the effect of increasing the number of biopsy cores in a FUS-Bx setting. One hundred and four Patients, most of whom had a repeat biopsy (82%), were included in this study. Tracy et al. demonstrated that the detection rate of csPCa increased from 26 to 44% by increasing the number of biopsy cores from one to three and again increased from 44 to 52% when the number of cores was increased to five per ROI [29].

We could only find three studies evaluating the value of additional biopsy cores in an MRI-Bx setting. Schimmöller et al. analyzed the MRI-targeted biopsy results of 771 lesions (290 patients) that were sampled with two cores as per their institution’s biopsy protocol to evaluate the feasibility of performing MRI-Bx with a single biopsy core. They reported an overall PCa detection rate of 50% (145/290) and a detection rate for clinically significant disease (defined as Gleason score ≥ 7 [4 + 3]) of 19% (54/290) on a patient level. The second biopsy core led to an upgrade from no cancer to any cancer (Gleason score ≥ 6 [3 + 3]) in 5% of all lesions sampled and to an upgrade from clinically insignificant cancer (6 [3 + 3]) to clinically significant cancer (≥ 7 [4 + 3]) in 0.5% (upgrade from Gleason score 6 [3 + 3] to Gleason score ≥ 7 [3 + 4] in 2.6% ) of cases. In 89% of all lesions the second biopsy core did not show a histologic difference compared with the first core [16].

Although the data presented by Schimmöller et al. show high Gleason concordance between the first and the second core, the possibility that more clinically significant cancers could have been found by obtaining more samples per target cannot be discarded. This assumption is further supported by our data. Although the second biopsy core in our analysis led to any upgrade/ newly diagnosed cancer in only 8.4% (21/250) of cases and to an upgrade from no cancer or ciPCa to csPCa in only 2.8% (7/250) of cases, we would still have missed 12.5% (8/64) of all csPCa detected after obtaining five cores if we had aborted the biopsy after the second core.

A second study by Subramanian et al. analyzed the impact the number of biopsy cores had on the detection rate of csPCa and ciPCa. Four hundred and forty-three patients with 747 lesions identified on mpMRI that underwent MRI-Bx met eligibility criteria and were evaluated in this retrospective single-center analysis. The biopsy protocol involved three cores per lesion as a default and allowed for less or more cores to be taken at the interventional radiologist´s discretion. In total 2359 biopsy cores were obtained. In three patients only one core per lesion was obtained. Two, three, four or five cores per lesion were obtained in 744, 719, 137 and 11 cases (lesions), respectively. Subramanian et al. detected 468 PCas in total of which 322 were csPCas (GS ≥ 7[3 + 4]). Of these, 78% were detected by the first core. The second, third, fourth and fifth core revealed additional csPCa (not found by previous cores) in 13%, 8.1%, 0.6% and 0%, respectively [17].

Seyfried et al. analyzed how the number of biopsy cores and concomitant sampling of a second lesion impacts detection rates. In total 128 patients with 163 lesions were included in this study. As per their institution’s protocol, at least two cores were obtained per ROI. More cores could have been obtained as to the interventional radiologist’s discretion. In total 163 lesions were sampled with at least two cores, 121 lesion were sampled with at least three cores and 52 lesions were sampled with at least four cores. Seyfried et al. found that in the group, where at least two cores had been obtained, the second core led to any upgrade in 12.9% of cases and to an upgrade from no cancer or ciPCa to csPCa in 7.4% of cases. In the group that was biopsied with at least three cores, the third core led to any upgrade in 10.7% and to an upgrade from no cancer or ciPCa to csPCa in 4.1% of cases relative to the first two cores. Obtaining a fourth core led to only one upgrade (1.9%) from ISUP Grade Group 2 to ISUP Grade Group 3 [18].

In both studies the possibility that more clinically significant cancers could have been found by obtaining a higher number of biopsy cores from all lesions cannot be ruled out. Moreover, the fact that Subramanian et al. and Seyfried et al. decided to obtain more samples in some cases makes their results prone to bias since the decision to acquire more samples could well have been based on the lesions being more challenging to target (because of lesion diameter or location of the lesion).

Considering detection rates only, we therefore do not deem a reduction of the number of cores per ROI preferable. But complications and adverse events might also play a role in determining the optimal number of biopsy cores. It is worth mentioning at this point that, overall, prostate biopsy is usually well tolerated and the most frequently occurring complications include cases of hematuria, hematospermia and hematochezia. Infectious complications occur less often but seem to have increased in the last decades [30]. According to a review by Borghesi et al., hematuria following prostate biopsy occurs in 2–84% of cases but is usually self-limiting or can be easily treated [30]. Moreover, hematuria was not perceived as a major or moderate problem by the majority of patients in a study by Rosario et al. [31]. Whether prevalence or duration of hematuria is affected by the number of cores is a matter of debate. Ghani et al. reported no significant differences in self-reported hematuria between groups that underwent systematic biopsies with 6, 8, or 12 cores [32]. Chowdhury et al. on the other hand concluded that the bleeding risk increases significantly with the number of biopsy cores [33]. Rectal bleeding was reported in 1.3–45% of cases according to Loeb et al. [34]. As is the case for hematuria, hematochezia is usually self-limiting and seldomly requires medical intervention. Rectal bleeding was significantly correlated with the number of biopsy cores obtained in the study by Ghani et al. [32], but not in the ERSPC trial [35]. It was not conceived as a major or moderate problem by most patients in the study by Rosario et al. [31]. Reported rates for hematospermia range between 1.1% and 92.6% [30]. Ghani et al. did not find a significant correlation between this kind of bleeding complication and the number of biopsy cores applied [32], whereas hematospermia was the only complication that was correlated to the number of biopsy cores in a study by Pepe et al. [36]. Although hematospermia is probably the least severe of these complications, it was perceived as the most problematic bleeding complication by patients in the study by Rosario et al. Twenty-six-point-six% of patients rated hematospermia as a moderate or major problem on a four point Likert-scale (none, mild, moderate, major) as opposed to 6.2% for hematuria and 2.5% for hematochezia [31].

Infectious complications following prostate biopsy occur less frequently. According to Borghesi et al. the reported rates range between 1% and 17.5% depending on the study [30]. Most of these infections are self-limiting or can be treated in an outpatient setting. A small proportion of men, however, requires hospitalization or even intensive care for the treatment of urosepsis. As is the case for bleeding complications, the question whether or not the number of biopsy cores plays a role in the development of infectious complications remains a controversy. Kalalahti et al. compared rates of infectious complications between 12-core systematic biopsy and MRI-targeted biopsy with a mean of 3.7 cores per ROI and found that the number of cores was significantly correlated with the occurrence of urinary tract infections (UTI) [37]. On the other hand, a review of randomized controlled trials that included 11 studies which analyzed the effect of the number of cores on infectious complications could not find a significant correlation [38]. Furthermore, a recent study by Wegelin et al. that compared the complication rates of MRI-Bx (median number of cores: 2), COG-Bx + TRUS-Bx (median number of cores: 13) and FUS-Bx + TRUS-Bx (median number of cores: 14) did not show a significant difference between the groups in terms of infectious complications [15]. It is worth mentioning, that although Kalalahti et al. found a significant correlation between the number of cores and urine culture positive UTI, they could not show this effect for blood culture positive infections [37]. Other complications following prostate biopsy include voiding problems, urinary retention, and erectile dysfunction. The pathophysiology of these adverse events following prostate biopsy is not well understood and no connection to the number of biopsy cores could have been established [30].

### Strengths and limitations

The strength of this study clearly lies in the systematic approach. To our knowledge this is the first study analyzing the effect of the number of cores on detection rates using an “in-bore”-biopsy approach in which every lesion was biopsied with at least five systematically distributed cores. Our study is, however, not free of limitations. Firstly, our study has all the limitations that are inherent to the retrospective design. Secondly, no conclusion can be drawn as to the accuracy of MRI-Bx compared to other biopsy approaches since we did not follow-up biopsy-negative patients or compared the biopsy results to the final pathology of prostatectomy specimens in cases in which patients opted for a radical prostatectomy. Also, as data on complications were not available from our cohort, we had to rely on complication rates reported by others. Finally, the number of csPCas in our cohort was relatively small with only 64 csPCas in total.

## Conclusion

In the light of the conflicting results regarding a correlation between the number of cores and the incidence of complications as well as the fact that most complications following prostate biopsy are minor and self-limiting, we advocate obtaining five cores per lesion to maximize detection rates. If, however, future research should show a significant correlation between the number of biopsy cores and serious adverse events, in particular infectious complications, a reduction in the number of cores to three per lesion might be justifiable: this approach may still allow for the detection of about 95% of all csPCa detected by a five-core biopsy.

### Electronic supplementary material

Below is the link to the electronic supplementary material.

Supplementary Material 1

## Data Availability

All data supporting our results are included in this article and its supplementary materials.

## Declarations

### Duplicate publication

The results of this study have been previously published in form of a diploma thesis as per the requirements of the Medical University of Vienna. The thesis is written in German language and is available at: https://digital.obvsg.at/urn/urn:nbn:at:at-ubmuw:1-47363.

## Abbreviations

ADC: Apparent diffusion coefficient
ax.: Axial
CI: Confidence interval
ciPCa: Clinically insignificant prostate cancer
COG: Bx–cognitive fusion biopsy
cor.: Coronal
csPCa: Clinically significant cancer
DCE: Dynamic contrast enhanced
DR: Detection rate
DWI: Diffusion–weighted imaging
FOV: Field of view
FUS: Bx–MRI–TRUS fusion biopsy
GS: Gleason Score
GUPS: Genitourinary Pathology Society
HASTE: Half–Fourier Acquisition Single–shot Turbo Spin Echo
IQR: Interquartile range
ISUP: International Society of Urological Pathology
mpMRI: Multiparametric MRI
MRI: Magnetic resonance imaging
MRI: Bx–MRI–targeted “in–bore”–biopsy
paraax.: Paraxial
parasag.: Parasagittal
PCa: Prostate cancer
PI-RADS: Prostate Imaging–Reporting and Data System
ROI: Region of interest
sag.: Sagittal
TE: Time to echo
TR: Repetition time
TruFISP: True Fast Imaging with Steady–state free Precession
TRUS: Transrectal ultrasound
TRUS: Bx–systematic TRUS–guided biopsy
TSE: Turbo spin echo
T1w: T1–weighted images
T2w: T2–weighted images
UTI: Urinary tract infection
VIBE: Volumetric Interpolated Breath–hold Examination
